# NeuroSPFNet: Vision–Language Semantic Prior-Guided Frequency-Enhanced Network for Brain Tumor Segmentation in Multimodal MRI

**DOI:** 10.3390/s26154963

**Published:** 2026-08-05

**Authors:** Yantong Liu, Seong-Yoon Shin, Hyun-Ae Lee

**Affiliations:** Department of Computer Information Engineering, Kunsan National University, Gunsan 54150, Republic of Korea; lyt1994@kunsan.ac.kr

**Keywords:** brain tumor segmentation, multimodal MRI, vision–language model, clinical semantic prior, frequency enhancement, boundary-aware segmentation, multimodal medical imaging, deep learning

## Abstract

Accurate brain tumor segmentation in multimodal magnetic resonance imaging (MRI) scans is essential for quantitative neuro-oncology analysis, treatment planning, and longitudinal disease monitoring. However, automatic delineation remains challenging because glioma subregions exhibit heterogeneous appearance across T1-weighted (T1), contrast-enhanced T1-weighted (T1ce), T2-weighted (T2), and fluid-attenuated inversion recovery (FLAIR) sequences; weak and irregular lesion boundaries; and severe scale variation between edema, tumor core, and enhancing tumor regions. This paper presents NeuroSPFNet, a vision–language semantic prior-guided frequency-enhanced segmentation framework for tumor subregion delineation in multimodal MRI volumes. NeuroSPFNet integrates four complementary components: a Clinical Vision–Language Prior Component (CVLPC) for category-level semantic guidance; a Frequency-Enhanced Boundary Module (FEBM) for boundary-sensitive representation learning; a Multi-Scale Lesion-Dominated Fusion Module (MLDFM) for hierarchical lesion aggregation; and a Lesion-Semantic Boundary Consistency Loss (LSBCL) for jointly constraining region overlap, boundary quality, and semantic alignment. The task-specific contribution lies in the closed coupling of shared BraTS-region semantic prototypes, gated frequency responses, lesion-weighted multiscale features, and semantic-boundary supervision within one volumetric segmentation pipeline. On the Brain Tumor Segmentation (BraTS) 2021 benchmark, NeuroSPFNet achieved Dice scores of 91.94%, 88.84%, and 85.38% for whole tumor, tumor core, and enhancing tumor, respectively, with a mean Dice of 88.72% and a mean 95th-percentile Hausdorff distance (HD95) of 3.58 mm. Ablation studies show distinct effects across subregions and metrics: semantic priors primarily strengthen tumor-core and enhancing-tumor discrimination, whereas frequency and boundary constraints primarily reduce contour error. NeuroSPFNet also attains competitive performance under the current setting, with moderate computational overhead of 42.58 million parameters, 78.60 billion floating-point operations (GFLOPs), and 265 ms per volume.

## 1. Introduction

Multimodal magnetic resonance imaging (MRI) is central to neuro-oncologic imaging because different MRI sequences provide complementary tissue contrast and lesion information. In glioma assessment, T1-weighted (T1), contrast-enhanced T1-weighted (T1ce), T2-weighted (T2), and fluid-attenuated inversion recovery (FLAIR) sequences jointly reveal tumor morphology, edema, enhancing tissue, and surrounding anatomical context. Accurate segmentation of the whole-tumor (WT), tumor-core (TC), and enhancing-tumor (ET) regions supports quantitative analysis for treatment planning, radiotherapy target delineation, response monitoring, and imaging-biomarker extraction [1,2,3]. These targets preserve complementary imaging phenotypes: WT measures the complete tumor-associated abnormality, including edema; TC isolates the enhancing and non-enhancing/necrotic central tumor; and ET quantifies the contrast-enhancing viable tumor. Their separate delineation supports region-specific volumetry and reveals changes in edema, central necrosis, and enhancement that a single merged mask would obscure. Despite the clinical importance of this task, manual segmentation is time-consuming, expertise-dependent, and difficult to standardize across centers.

Automatic brain tumor segmentation from multimodal MRI scans remains challenging. First, tumor appearance is heterogeneous across patients, scanners, institutions, and imaging sequences. A tumor component may be visually prominent in one modality and weakly represented in another. Second, tumor boundaries are often ambiguous because infiltrative growth, edema, and imaging noise produce weak and irregular margins. Third, lesion subregions vary substantially in size: WT may cover a broad area, whereas ET may be small, fragmented, and heavily imbalanced relative to normal tissue. These challenges require lesion-aware representation, boundary-sensitive feature enhancement, and robust multiscale fusion.

NeuroSPFNet assigns one targeted component to each failure mode and couples the four components through a shared segmentation pathway. The Clinical Vision–Language Prior Component (CVLPC) converts MRI-oriented descriptions of WT, TC, ET, and edema into category prototypes that condition volumetric visual features, directly addressing subregions with overlapping appearance. The Frequency-Enhanced Boundary Module (FEBM) gates three-dimensional spectral responses to strengthen weak local transitions and preserve irregular lesion margins. The Multi-Scale Lesion-Dominated Fusion Module (MLDFM) reweights and aligns four decoder resolutions so that broad edema and small enhancing foci contribute within a common lesion-centered representation. The Lesion-Semantic Boundary Consistency Loss (LSBCL) jointly constrains region overlap, three-dimensional Sobel gradient agreement, and alignment between predicted lesion embeddings and the same category prototypes used by CVLPC. This allocation connects semantic ambiguity, boundary uncertainty, and scale imbalance to explicit, experimentally separable mechanisms.

Deep convolutional neural networks have become standard tools for biomedical segmentation. U-Net and its variants use encoder–decoder structures and skip connections to recover spatial detail [4,5,6,7]. Transformer-based segmentation networks further improve global context modeling through self-attention and hierarchical token representation [8,9,10,11]. Recent semantic-prior, boundary-aware, and frequency-domain studies demonstrate the value of these complementary signals [12,13,14,15]. A remaining task-level gap is the coordinated use of subregion semantics, boundary-frequency responses, and scale-dependent lesion evidence within one BraTS-oriented optimization pathway.

This study proposes NeuroSPFNet, a vision–language semantic prior-guided frequency-enhanced network for brain tumor segmentation in multimodal MRI volumes. The method uses one shared set of WT-, TC-, ET-, and edema-oriented semantic prototypes to condition intermediate visual features and to supervise class-specific output embeddings. CVLPC, FEBM, and MLDFM form the feature pathway, and LSBCL supplies region, contour, and semantic supervision at its output.

The main contributions are as follows:

- We introduce a shared-prototype semantic pathway that uses the same BraTS-oriented descriptors for intermediate visual conditioning and output-level semantic supervision.
- We couple semantic conditioning with gated volumetric frequency enhancement and lesion-weighted multiscale fusion to address subregion ambiguity, weak margins, and lesion-scale imbalance.
- We formulate LSBCL to coordinate region overlap, three-dimensional boundary consistency, and class-semantic alignment.
- We evaluate the contribution of each link through component, semantic-prior, frequency-strategy, and loss-term ablations.

## 2. Related Work

The review of the literature is organized starting with single-sequence segmentation through to multimodal and transformer-based models, followed by semantic-prior and boundary-frequency methods. This progression identifies the three linked requirements addressed by NeuroSPFNet: subregion discrimination, weak-boundary recovery, and scale-aware lesion aggregation.

### 2.1. Medical Image Segmentation

Convolutional neural network (CNN)-based biomedical segmentation methods remain highly competitive because they preserve local spatial detail and can be trained efficiently with limited data. U-Net introduced the encoder–decoder structure with skip connections [4], three-dimensional (3D) U-Net extended dense prediction to volumetric input [5], and Attention U-Net used attention gates to reduce irrelevant responses [6]. nnU-Net showed that robust preprocessing, training configuration, and postprocessing can produce strong general segmentation performance [7]. Single-sequence models establish strong spatial baselines, while their input cannot represent the distinct enhancement, edema, and tissue-relaxation information distributed across the four BraTS MRI sequences. This motivates the use of a stable U-Net-like backbone as the base architecture.

### 2.2. Brain Tumor Segmentation in Multimodal MRI

The Brain Tumor Segmentation (BraTS) benchmark has provided a widely used setting for evaluating glioma segmentation methods [1,2,3,16]. Typical pipelines use T1, T1ce, T2, and FLAIR sequences and report Dice and the 95th-percentile Hausdorff distance (HD95) for WT, TC, and ET. T1 provides anatomical context, T1ce highlights enhancing tissue, T2 depicts water-rich abnormalities, and FLAIR suppresses the cerebrospinal-fluid signal to emphasize edema and infiltrative change. CNN-based brain tumor segmentation has explored cascaded networks, multiscale processing, residual learning, and volumetric context [17,18,19,20]. Nevertheless, accurately segmenting small enhancing regions and fuzzy boundaries under inter-modality heterogeneity remains difficult.

### 2.3. Transformer-Based Medical Image Segmentation

Transformers model long-range dependency and have been adapted to segmentation through hybrid and hierarchical designs [21,22]. TransUNet combines CNN feature extraction with transformer encoding [8]; Swin-Unet uses shifted window attention for U-shaped segmentation [9]; and UNETR uses transformer encoders for 3D medical segmentation [10]. Recent volumetric models, including nnFormer and Swin UNETR, further strengthen hierarchical 3D context modeling [11,23]. Their progress sharpens the remaining need for explicit lesion-category conditioning and boundary-focused optimization.

### 2.4. Vision–Language Semantic Priors in Medical Imaging

Vision–language models align visual features with text descriptions and have shown broad transfer capability, exemplified by Contrastive Language–Image Pre-training (CLIP) [24]. Biomedical language and image–text models, including BioBERT, PubMedBERT, ClinicalBERT, ConVIRT, GLoRIA, MedCLIP, and BioMedCLIP, provide useful representations for medical text or medical image–text data [25,26,27,28,29,30,31]. NeuroSPFNet narrows this general paradigm to four BraTS-oriented prototypes and reuses them in both feature conditioning and output-level semantic consistency.

### 2.5. Frequency-Domain and Boundary-Aware Feature Enhancement

Boundary quality is critical in medical segmentation because clinical structures often have irregular contours. Boundary loss, Hausdorff-inspired loss, surface-aware objectives, and edge consistency constraints have been proposed to improve contour accuracy [14,15,32]. Frequency-domain analysis provides a complementary view of local transitions and high-frequency image detail [33,34,35]. NeuroSPFNet links gated three-dimensional frequency responses to Sobel gradient boundary consistency, while semantic conditioning and multiscale lesion weighting address class ambiguity and scale variation in the same pathway.

## 3. Materials and Methods

### 3.1. Overview of NeuroSPFNet

**Figure 1:**
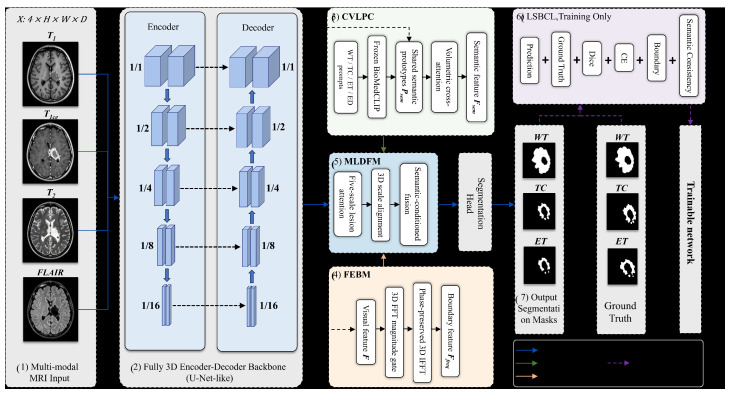
Three-dimensional NeuroSPFNet and information flow. Four co-registered MRI sequences enter the five-resolution 3D encoder–decoder. Backbone features supply the visual queries for CVLPC and the spectral input for FEBM; the resulting semantic and frequency-enhanced features join four selected decoder resolutions in MLDFM before the segmentation head predicts WT, TC, and ET. Blue, green, and orange solid arrows denote visual, semantic, and frequency-enhanced feature flow, respectively. Black dashed arrows denote encoder–decoder skip connections or auxiliary backbone feature inputs, and purple dashed arrows denote training-only LSBCL supervision.

NeuroSPFNet is implemented as a three-dimensional volumetric network. Let $X\in R^{4\times H\times W\times D}$ denote four co-registered MRI sequences, T1, T1ce, T2, and FLAIR, represented as the four input planes of the computational tensor. The term “channel” is used only for this tensor dimension; each MRI sequence measures distinct tissue contrast and physiology. A 2.5D model would stack a target slice with adjacent slices and apply two-dimensional operators, whereas the reported model uses three-dimensional convolutions, 128×128×128 subvolumes, and full-volume sliding-window inference. NeuroSPFNet augments a U-Net-like encoder–decoder with CVLPC, FEBM, MLDFM, and LSBCL, as shown in Figure 1. The overview displays all five backbone resolutions; MLDFM uses four selected decoder resolutions for the aligned fusion detailed below.

### 3.2. Clinical Vision–Language Prior Component

CVLPC constructs semantic priors from concise clinical descriptions of tumor subregions. The prompts describe WT, TC, ET, and edema using MRI-relevant language. A frozen text encoder $\Phi_{\mathrm{text}}$ produces embeddings for the prompt set $T=\{t_{\mathrm{WT}},t_{\mathrm{TC}},t_{\mathrm{ET}},t_{\mathrm{ED}}\}$. A trainable projection aligns the text embedding dimension to the visual feature dimension:

(1) $$P_{\mathrm{sem}}=W_{p}\Phi_{\mathrm{text}}\left(T\right)+b_{p},$$

where $P_{\mathrm{sem}}\in R^{C_{s}\times d}$ contains semantic prototypes for tumor-related categories.

The exact prompts used to construct the four category prototypes were as follows:

- WT: “A whole-tumor region on multimodal brain MRI, including peritumoral edema, non-enhancing or necrotic tumor core, and enhancing tumor.”
- TC: “A tumor-core region on multimodal brain MRI, including enhancing tumor and non-enhancing or necrotic core, while excluding peritumoral edema.”
- ET: “An enhancing-tumor region showing contrast enhancement on post-contrast T1-weighted MRI within the tumor core.”
- ED: “A peritumoral edema region showing hyperintensity on T2-weighted and FLAIR MRI around the tumor core.”

BioMedCLIP [31] was used as the frozen text encoder. Its parameters remained fixed, and only the semantic projection and downstream segmentation network were optimized.

**Figure 2:**
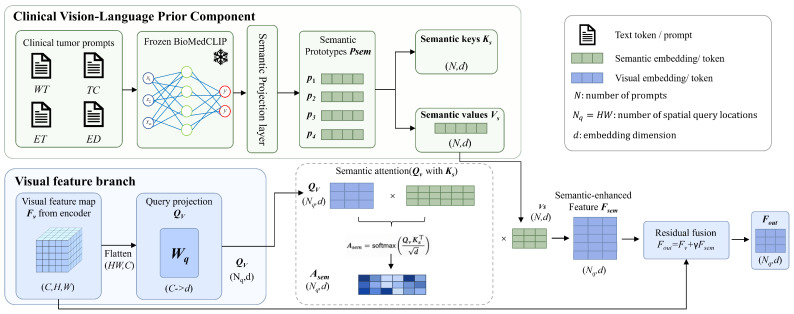
Clinical Vision–Language Prior Component (CVLPC). The four WT, TC, ET, and ED prompts are encoded by frozen BioMedCLIP and projected into shared semantic prototypes. The planar token arrangement is used only to keep the schematic legible; in the implemented volumetric branch, $F_{v}\in R^{C\times H\times W\times D}$ is flattened into $N_{q}=HWD$ visual queries. The prototypes act as semantic keys and values in cross-attention, followed by residual fusion with the 3D visual features.

For an intermediate visual feature $F_{v}$, visual queries and semantic keys/values are computed by linear projections. Specifically, the volumetric feature is rearranged from C×H×W×D to $N_{q}\times C$, where $N_{q}=HWD$; the HW layout in Figure 2 is a compact graphical projection of this volumetric tokenization. Semantic attention is defined as follows:

(2) $$A_{\mathrm{sem}}=\mathrm{softmax}\left(\frac{Q_{v}K_{s}^{⊤}}{\sqrt{d}}\right),\;F_{\mathrm{sem}}=A_{\mathrm{sem}}V_{s},\;F_{\mathrm{out}}=F_{v}+\gamma F_{\mathrm{sem}}.$$

This design introduces category-level prior guidance while keeping image evidence as the dominant segmentation signal.

### 3.3. Frequency-Enhanced Boundary Module

FEBM targets weak and irregular lesion boundaries by enhancing high-frequency feature responses. Given feature map *F*, the fast Fourier transform (FFT)-based branch computes a spectral representation, learns a frequency gate, and reconstructs an enhanced response:

(3) $$\hat{F}=F\left(F\right),\;G=\sigma (\mathrm{Conv}(|\hat{F}|)),\;F_{\mathrm{freq}}=F^{-1}\left(\hat{F}⊙G\right),\;F_{\mathrm{FEBM}}=F+\alpha F_{\mathrm{freq}}.$$

The Fourier transform is applied along the three spatial axes (H,W,D) with orthonormal normalization. The real-valued gate is generated from the spectral magnitude by a 1×1×1 convolution and sigmoid activation, then multiplied with the complex spectrum so that the original phase is retained. The real component of the inverse transform is fused with the spatial residual through the trainable coefficient α; frequency shifting is used only for spectrum visualization. For efficient ablation, a high-frequency residual approximation can also be used:

(4) $$F_{\mathrm{high}}=F-\mathrm{Up}\left(\mathrm{AvgPool}\left(F\right)\right),\;F_{\mathrm{FEBM}}=F+\alpha \mathrm{Conv}\left(F_{\mathrm{high}}\right).$$

**Figure 3:**
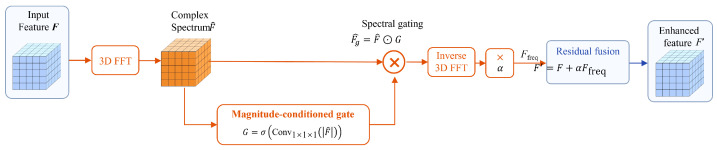
Frequency-Enhanced Boundary Module (FEBM). A 3D FFT is applied along the *H*, *W*, and *D* axes. A 1×1×1 convolution predicts a real-valued gate from the spectral magnitude; this gate modulates the complex spectrum while retaining phase. The real component reconstructed by the inverse 3D FFT is scaled by the learnable coefficient α and added to the unchanged spatial residual *F*, yielding $F_{\mathrm{FEBM}}=F+\alpha F_{\mathrm{freq}}$. Orange arrows denote frequency-domain transformation, gating, inverse transformation, and residual scaling; the blue arrow denotes the enhanced spatial-feature output.

Figure 3 summarizes the frequency-domain gating and residual reconstruction pathway.

### 3.4. Multi-Scale Lesion-Dominated Fusion Module

MLDFM fuses hierarchical features while emphasizing lesion-relevant regions. Four decoder resolutions are selected from the five-stage backbone. For features ${\left\{F_{i}\right\}}_{i=1}^{4}$, lesion attention maps are computed and used for three-dimensional scale-aligned fusion:

(5) $$A_{i}=\sigma \;\left(\mathrm{Conv}3D_{3\times 3\times 3}\left(F_{i}\right)\right),\;{\hat{F}}_{i}=\mathrm{Align}3D\left(A_{i}⊙F_{i}\right),\;F_{\mathrm{MLDF}}=\sum_{i=1}^{4}{\hat{F}}_{i}.$$

Scale 1 defines the reference resolution $\left(H_{1},W_{1},D_{1}\right)$; scales 2–4 are upsampled to this spatial size, and a channel-alignment projection produces a common dimension $C_{a}$. Semantic conditioning is then applied through cross-attention:

(6) $$F_{\mathrm{sem}}=\mathrm{CrossAttention}\left(Q=F_{\mathrm{MLDF}},K=K_{s},V=V_{s}\right),\;F_{\mathrm{MLDFM}}=F_{\mathrm{MLDF}}+\beta F_{\mathrm{sem}}.$$

**Figure 4:**
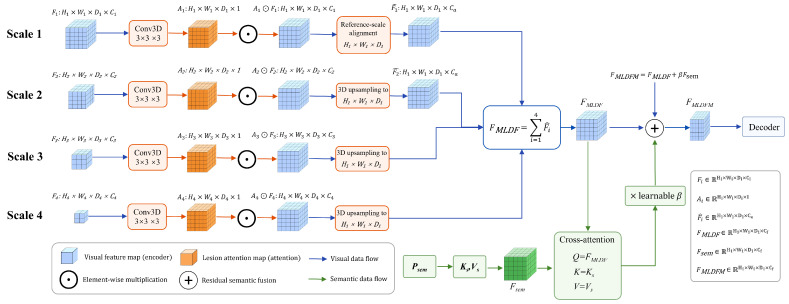
Multi-Scale Lesion-Dominated Fusion Module (MLDFM). Four volumetric decoder features are reweighted by $A_{i}=\sigma \left(\mathrm{Conv}3D_{3\times 3\times 3}\left(F_{i}\right)\right)$, aligned to $\left(H_{1},W_{1},D_{1}\right)$, and fused exclusively by element-wise summation. Cross-attention uses $F_{\mathrm{MLDF}}$ as the visual query and the shared CVLPC prototypes as semantic keys and values. The learnable residual coefficient β produces $F_{\mathrm{MLDFM}}=F_{\mathrm{MLDF}}+\beta F_{\mathrm{sem}}$ before decoder integration. Orange arrows denote lesion-attention map generation and feature reweighting, blue arrows denote multiscale visual-feature alignment and fusion, and green arrows denote semantic conditioning and its gated residual pathway.

Figure 4 summarizes scale alignment, lesion-attention weighting, element-wise fusion, and semantic conditioning.

### 3.5. Lesion-Semantic Boundary Consistency Loss

The optimization objective combines Dice loss, cross-entropy loss, boundary consistency loss, and semantic consistency loss. For each tumor-related class *c*, the predicted lesion embedding $e_{c}^{\mathrm{pred}}$ is aligned with the semantic prior $p_{c}^{\mathrm{sem}}$:

(7) $$L_{\mathrm{sem}}=1-\frac{1}{C}\sum_{c=1}^{C}\frac{e_{c}^{\mathrm{pred}}\cdot p_{c}^{\mathrm{sem}}}{∥e_{c}^{\mathrm{pred}}{∥}_{2}{∥p_{c}^{\mathrm{sem}}∥}_{2}}.$$

Boundary consistency is computed from fixed three-dimensional Sobel gradients. For class *c*, prediction probability $P_{c}$, and binary reference $G_{c}$, the implemented term is

(8) $$L_{\mathrm{boundary}}=\frac{1}{C}\sum_{c=1}^{C}\frac{1}{N}\left(∥S_{x}\left(P_{c}\right)-S_{x}\left(G_{c}\right){∥}_{1}+∥S_{y}\left(P_{c}\right)-S_{y}\left(G_{c}\right){∥}_{1}+{∥S_{z}\left(P_{c}\right)-S_{z}\left(G_{c}\right)∥}_{1}\right),$$

where $S_{x}$, $S_{y}$, and $S_{z}$ are fixed Sobel kernels and *N* is the number of voxels. The full objective is

(9) $$L_{\mathrm{total}}=\lambda_{1}L_{\mathrm{Dice}}+\lambda_{2}L_{\mathrm{CE}}+\lambda_{3}L_{\mathrm{boundary}}+\lambda_{4}L_{\mathrm{sem}}.$$

### 3.6. Implementation Details

The implementation was developed using PyTorch version 2.1.2 (Meta Platforms, Inc., Menlo Park, CA, USA) and optimized with AdamW using an initial learning rate of $1\times 10^{-4}$ and cosine learning-rate decay. The five-stage 3D encoder used channel widths of 32, 64, 128, 256, and 512. Each stage contained two 3×3×3 convolutions with instance normalization and Leaky ReLU activation. Downsampling used stride-2 convolution; each decoder stage used transposed convolution, concatenation with the corresponding encoder feature, and two convolutional blocks. The prediction head produced three sigmoid region maps for WT, TC, and ET. All experiments were trained for 300 epochs with a batch size of 2 on two NVIDIA GeForce RTX 4090 graphics processing units (NVIDIA Corporation, Santa Clara, CA, USA) under deterministic random seeds. Input volumes were cropped into foreground-centered patches of size 128×128×128, and sliding-window inference with a 50% overlap was used during evaluation. Random flipping was applied independently along each spatial axis with probability 0.5. Random rotation was sampled uniformly from −15° to 15°, and Gaussian noise with a standard deviation sampled from 0 to 0.1 was applied with probability 0.15. The loss weights were empirically set to $\lambda_{1}=1.0$, $\lambda_{2}=1.0$, $\lambda_{3}=0.5$, and $\lambda_{4}=0.2$.

### 3.7. Dataset and Preprocessing

The experiments used the 1251 labeled cases released as the BraTS 2021 training cohort [16]. Each case contains co-registered T1, T1ce, T2, and FLAIR sequences and an organizer-provided reference segmentation. We included all cases with the four required sequences and a valid reference mask; no case was excluded after the integrity check. WT was formed from BraTS labels 1, 2, and 4, TC from labels 1 and 4, and ET from label 4. Reference masks were produced and quality-controlled by the BraTS data provider under the benchmark annotation protocol. The official BraTS 2021 challenge page (https://www.synapse.org/Synapse:syn25829067/wiki/626944) was accessed on 28 July 2026.

A patient-level random partition assigned 1000 labeled cases to training and 251 labeled cases to held-out internal evaluation. The official 219-case BraTS 2021 validation cohort was not used for local quantitative evaluation because its reference labels are not publicly available. The partition and preprocessing protocol are summarized in Table 1. All internally generated quantitative values were computed on the 251-case held-out subset; the two explicitly labeled published-reference rows in Table 2 retain their source-reported evaluation settings. BraTS-released volumes had undergone organizer-provided co-registration, 1 mm isotropic resampling, and skull stripping. We applied no additional N4 bias-field correction; each sequence was normalized by z-score within the nonzero brain mask.

### 3.8. Evaluation Metrics

The Dice score and the 95th-percentile Hausdorff distance (HD95) were used for WT, TC, and ET. Dice measures volumetric overlap:

(10) $$\mathrm{Dice}=\frac{2TP}{2TP+FP+FN}.$$

HD95 is the 95th percentile of the bidirectional Euclidean surface distances between the predicted and reference masks. The distances were computed in physical space using the Neuroimaging Informatics Technology Initiative (NIfTI) voxel spacing and reported in millimeters. For every internally evaluated model and ablation configuration, the patient-level score vector contained n=251 observations. Two-sided 95% confidence intervals (CIs) for the patient-level mean were calculated using Student’s *t* interval:

(11) $${\mathrm{CI}}_{95\%}=\bar{x}\pm t_{0.975,n-1}\frac{s}{\sqrt{n}},$$

where $\bar{x}$ is the patient-level sample mean, *s* is the sample standard deviation calculated with n−1 degrees of freedom, and $t_{0.975,250}=1.96949776$. Table 2, Table 3, Table 4, Table 5 and Table 6 report mean [95% CI]. CIs are unavailable for the two published-reference rows in Table 2 because their source studies did not provide patient-level predictions or score vectors. The intervals quantify uncertainty in the reported means; no pairwise hypothesis test is claimed.

### 3.9. Experimental Protocol

All reproduced models used the same 1000/251 patient partition and evaluation script. The nnU-Net-style comparator was trained under this unified protocol and is identified as such in Table 2; it does not represent the complete native self-configuring nnU-Net pipeline. Published nnFormer and Swin UNETR results are reported separately as BraTS 2021 reference values because their evaluation partitions and training recipes differ from the present internal protocol. Table 3 adds CVLPC, FEBM, MLDFM, and LSBCL sequentially to the same baseline. Table 4 changes only the semantic prior source; Table 5 changes only the frequency-enhancement strategy; and Table 6 changes only the active loss terms; all remaining settings are held fixed.

## 4. Experiments and Results

### 4.1. Main Comparison

Table 2 separates models reproduced on the 251-case held-out subset from published BraTS 2021 results reported under source-specific protocols. Within the reproduced block, NeuroSPFNet yielded a mean Dice of 88.72% [87.10%, 90.34%] and a mean HD95 of 3.58 mm [2.46 mm, 4.70 mm]. The widest class-specific interval occurred for ET Dice, 85.38% [82.52%, 88.24%], reflecting greater interpatient variability for the smallest tumor subregion. The nnU-Net-style comparator used the unified study protocol and is identified separately from the complete native self-configuring nnU-Net pipeline. Published nnFormer and Swin UNETR values position the method against recent volumetric transformers under source-specific evaluation settings. Their CIs remain unavailable because the source studies did not release patient-level predictions or score vectors. Within the unified block, overlapping CIs identify several close comparisons and support interpreting the reported ordering as point-estimate evidence rather than a formal pairwise significance result.

### 4.2. Ablation Study

The ablation protocol isolates the contribution of each proposed component. Table 3 evaluates CVLPC, FEBM, MLDFM, and LSBCL. Table 4 compares the semantic prior sources. Table 5 evaluates the frequency strategies. Table 6 evaluates the loss components. Each ablation changes only the factor named in the corresponding table while retaining the same patient partition, optimizer, training duration, inference procedure, and evaluation script.

The component-ablation results in Table 3 show complementary numerical patterns across the four modules. Relative to the baseline mean Dice of 85.16% [83.30%, 87.02%], the CVLPC configuration reached 86.84% [85.10%, 88.58%], with the largest point-estimate changes in TC and ET. Adding FEBM increased the mean Dice to 87.38% [85.68%, 89.08%] and reduced the mean HD95 to 4.28 mm [3.14 mm, 5.42 mm], while WT Dice remained stable. MLDFM increased the mean Dice to 88.01% [86.36%, 89.66%]. The full configuration reached 88.72% [87.10%, 90.34%] mean Dice and 3.58 mm [2.46 mm, 4.70 mm] mean HD95. The intervals expose substantial patient-level variability, while the sequential point estimates provide a consistent Dice increase and HD95 reduction across the complete component pathway.

Table 4 shows distinct patterns across prior sources. Random embeddings produced a mean Dice of 85.22% [83.37%, 87.07%], close to the no-prior configuration at 85.16% [83.30%, 87.02%]. Learned embeddings and the generic CLIP text encoder produced intermediate point estimates. BioClinicalBERT and BioMedCLIP produced the strongest numerical results in this ablation, with BioMedCLIP reaching 86.84% [85.10%, 88.58%] mean Dice and 4.95 mm [3.74 mm, 6.16 mm] mean HD95. The concentration of the point-estimate gains in TC and ET is consistent with the proposed role of clinically aligned priors in tumor-subregion discrimination.

The frequency enhancement ablation in Table 5 places fast Fourier transform (FFT) frequency gating at the strongest observed Dice and HD95 operating point. Sobel edge enhancement yielded 87.05% [85.34%, 88.76%] mean Dice and 4.40 mm [3.24 mm, 5.56 mm] mean HD95, while the high-frequency residual strategy reached 87.47% [85.78%, 89.16%] and 4.52 mm [3.35 mm, 5.69 mm]. Relative to the non-frequency-enhanced setting, FFT gating changed mean Dice from 86.84% [85.10%, 88.58%] to 87.56% [85.88%, 89.24%] and mean HD95 from 4.95 mm [3.74 mm, 6.16 mm] to 4.15 mm [3.03 mm, 5.27 mm].

The loss ablation in Table 6 shows distinct metric responses across the loss terms. Boundary loss reduced mean HD95 from 4.16 mm [3.03 mm, 5.29 mm] to 3.70 mm [2.62 mm, 4.78 mm]. Semantic loss produced the strongest ET Dice point estimate among the single-added-loss settings, reaching 85.31% [82.43%, 88.19%], with a mean Dice of 88.46% [86.83%, 90.09%]. The full LSBCL configuration reached the highest observed mean Dice, 88.72% [87.10%, 90.34%], and the lowest observed mean HD95, 3.58 mm [2.46 mm, 4.70 mm]. The overlapping intervals quantify the scale of patient-level uncertainty, while the metric-specific point-estimate pattern aligns with the designated role of each loss term.

### 4.3. Visualization Analysis

**Figure 5:**
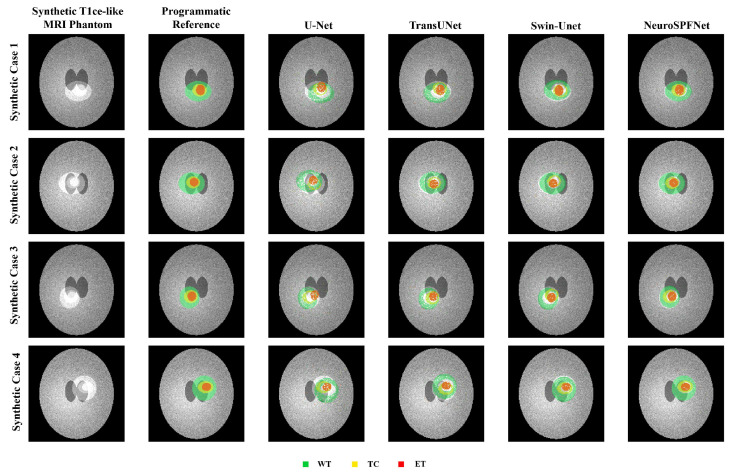
Controlled segmentation stress test on synthetic MRI-like phantoms. Each row shows a synthetic T1ce-like slice, the programmatic reference mask, and predictions from U-Net, TransUNet, Swin-Unet, and NeuroSPFNet. These phantoms were used only for qualitative visualization and were excluded from every BraTS quantitative result.

**Figure 6:**
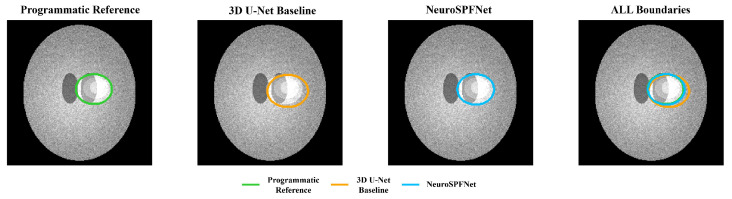
Boundary stress test on a synthetic T1ce-like MRI phantom. Green, orange, and cyan contours denote the programmatic reference, 3D U-Net baseline prediction, and NeuroSPFNet prediction, respectively. The panel isolates contour behavior under controlled contrast and noise and does not show a patient scan.

**Figure 7:**
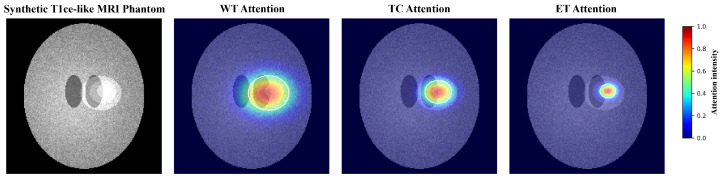
Class-specific attention on a synthetic T1ce-like MRI phantom. Heat maps show normalized WT, TC, and ET responses on controlled nested-lesion geometry; white contours denote the programmatic class boundaries. The phantom was used exclusively for qualitative inspection.

Figure 5, Figure 6 and Figure 7 present controlled qualitative stress tests on programmatically generated two-dimensional MRI-like phantoms. The phantoms contain nested WT, TC, and ET regions with controlled intensity heterogeneity, boundary irregularity, spatial displacement, and Gaussian noise. NeuroSPFNet and all comparators were trained exclusively on the 1000-patient BraTS training partition. The phantoms were generated after training and were excluded from parameter optimization, model selection, quantitative evaluation, and every BraTS result in Table 2, Table 3, Table 4, Table 5 and Table 6. Their simplified geometry supplies exact programmatic masks and controlled perturbations, allowing segmentation leakage, contour displacement, and class-specific response localization to be inspected without anatomical and acquisition factors changing simultaneously. The resulting panels provide mechanism-oriented stress tests that complement the patient-level quantitative evaluation; they carry no estimate of in vivo performance.

Figure 5 tests whether each network preserves the nested WT–TC–ET organization across four changes in lesion position, surrounding intensity, and noise. U-Net produces the most fragmented masks and scattered false-positive voxels, while TransUNet and Swin-Unet recover the principal lesion geometry but retain local leakage and uneven subregion boundaries. NeuroSPFNet yields the closest visual correspondence to the programmatic masks across the four cases, particularly for the compact ET center and the continuity of the surrounding TC and WT regions. This pattern is consistent with the quantitative ablations: category prototypes support nested subregion discrimination, and lesion-weighted multiscale fusion stabilizes regions with markedly different spatial extent.

Figure 6 isolates outer-contour behavior in a case with an irregular, low-contrast margin. The 3D U-Net contour shows a visible outward displacement along the inferior and right margins. The NeuroSPFNet contour follows the programmatic boundary more closely over most of the circumference and preserves the asymmetric lesion shape. The localized reduction in contour deviation provides a visual mechanism-level counterpart to the lower HD95 observed after FEBM and boundary-consistency supervision in Table 5 and Table 6.

Figure 7 examines whether the shared semantic prototypes produce spatially ordered class responses. WT attention covers the full abnormal region, TC attention contracts toward the central tumor compartment, and ET attention forms the most compact response around the enhancing center. The progressive reduction in response extent follows the programmed containment relationship ET⊂TC⊂WT, while activation outside the lesion remains low. This class-dependent localization supports the proposed role of CVLPC and the semantic term in separating nested tumor targets with overlapping image evidence.

### 4.4. Computational Efficiency

Table 7 reports parameter count, billions of floating-point operations (GFLOPs), inference time, and mean Dice. NeuroSPFNet achieved the highest mean Dice in this comparison with moderate overhead. Specifically, the model required 42.58 M parameters, 78.60 GFLOPs, and 265 ms per volume, making it slower and larger than the nnU-Net-style 3D U-Net comparator while still being more compact than TransUNet and UNETR.

## 5. Discussion

CVLPC introduces clinical semantic priors as category-level guidance for tumor subregions. The semantic-prior point estimates place random embeddings close to the no-prior setting, learned embeddings above that reference, and the biomedical text–image priors at the strongest observed Dice–HD95 operating points. The numerical pattern is concentrated in TC and ET, where visual ambiguity and class imbalance are common. The shared prototypes influence both intermediate feature selection and output embedding consistency, which provides a mechanism-level explanation for the observed concentration in subregion discrimination. The semantic branch supplies category-level descriptors for computer-assisted segmentation and does not perform diagnostic reasoning.

FEBM is motivated by the weak and irregular boundaries observed in MRI tumor segmentation. In the frequency ablation, FFT-based gating changed the mean HD95 point estimate from 4.95 mm to 4.15 mm and the mean Dice point estimate from 86.84% to 87.56%. Sobel filtering produced its clearest numerical change in boundary distance, and high-frequency residual enhancement produced a Dice point estimate close to FFT with a higher HD95 point estimate. The alignment between these descriptive results and FEBM’s boundary-targeted construction supports continued evaluation of gated high-frequency responses for lesion-margin delineation. The larger change in HD95 than in Dice is consistent with FEBM’s operating target: the gate emphasizes spectral responses associated with local transitions, while the Sobel gradient term suppresses isolated contour deviations.

MLDFM addresses the scale gap between large edema regions and small enhancing components. In the progressive ablation, introducing MLDFM after CVLPC and FEBM changed the mean Dice point estimate from 87.38% to 88.01% and the mean HD95 point estimate from 4.28 mm to 4.16 mm. The stronger numerical change in overlap than in boundary distance is consistent with a scale-sensitive regional representation across heterogeneous lesion sizes.

The observed numerical pattern across WT, TC, and ET follows the structure of the task. WT is larger and strongly expressed on T2/FLAIR, leaving less headroom for semantic disambiguation. TC requires separation of edema from central tumor tissue, while ET is small, contrast-dependent, and strongly imbalanced. CVLPC produces its largest observed changes in TC and ET, FEBM produces its clearest change in boundary distance, and MLDFM supplies the scale context needed to connect small enhancing regions with the broader lesion hierarchy.

NeuroSPFNet provides automated WT, TC, and ET measurements for research-oriented quantitative analysis. Its present evidence establishes benchmark-level segmentation performance; clinical workflow benefit requires dedicated external and prospective evaluation.

This study has five principal limitations. Evaluation is restricted to one public benchmark and one internal 251-patient partition; the reported 95% CIs quantify patient-level uncertainty within this partition, and several close model comparisons have overlapping intervals. The four semantic prompts are manually specified and remain sensitive to wording and text-encoder choice. The controlled synthetic phantoms inspect mechanism behavior but do not represent the diversity of in vivo MRI. The semantic and spectral branches add computation relative to the base network. External multi-center validation, paired model-comparison analysis on prospectively retained score vectors, missing-sequence testing, prompt robustness, uncertainty calibration, and workflow-level evaluation define the next validation stage.

## 6. Conclusions

This work presents NeuroSPFNet, a vision–language semantic prior-guided frequency-enhanced framework for brain tumor segmentation in multimodal MRI volumes. By combining CVLPC, FEBM, MLDFM, and LSBCL, the proposed method targets tumor-subregion discrimination, boundary delineation, and multiscale lesion representation. On the 251-case held-out BraTS 2021 subset, NeuroSPFNet yielded Dice scores of 91.94% [90.70%, 93.18%], 88.84% [86.85%, 90.83%], and 85.38% [82.52%, 88.24%] for WT, TC, and ET, respectively, with a mean Dice of 88.72% [87.10%, 90.34%] and a mean HD95 of 3.58 mm [2.46 mm, 4.70 mm]. The component, prior-source, frequency-strategy, and loss-term ablations produce complementary patterns across subregion discrimination and contour accuracy, with 95% CIs reporting the patient-level uncertainty of every internally evaluated score. NeuroSPFNet also presents moderate computational overhead under the current setting. The results position the coupling of shared semantic prototypes, frequency-enhanced boundary modeling, and multiscale lesion fusion as a testable benchmark contribution. External multi-center validation and paired model-comparison analysis define the next evaluation stage.

## Figures and Tables

**Table 1:** BraTS 2021 partition and preprocessing protocol.

Dataset	MRI Sequences	Task	Training	Held-Out Evaluation	Preprocessing
BraTS 2021	T1, T1ce, T2, FLAIR	Brain tumor multi-class segmentation	1000	251	Organizer-provided co-registration, 1 mm isotropic resampling and skull stripping; no additional N4 correction; nonzero-mask z-score normalization; 128×128×128 foreground-centered patches; flipping, rotation, and Gaussian noise augmentation

**Table 2:** Comparison with models reproduced under the unified study protocol and published BraTS 2021 results. Cells report mean [95% confidence interval]. HD95 values are in millimeters. Confidence intervals are reported for all models evaluated under the unified 251-patient protocol. N/A indicates that confidence intervals are unavailable for the two published-reference rows because patient-level predictions and score vectors were not provided by the source studies. Boldface is limited to the reproduced-model block and denotes the best point estimate within that block.

Method	Backbone	WT Dice	TC Dice	ET Dice	Mean Dice	WT HD95	TC HD95	ET HD95	Mean HD95
*CNN-based methods*									
U-Net	CNN	89.24 [87.81, 90.67]	83.56 [81.07, 86.05]	78.45 [74.79, 82.11]	83.75 [81.64, 85.86]	5.84 [4.70, 6.98]	7.32 [5.46, 9.18]	4.95 [2.71, 7.19]	6.04 [4.67, 7.41]
Attention U-Net	CNN	89.85 [88.47, 91.23]	84.42 [82.03, 86.81]	79.18 [75.64, 82.72]	84.48 [82.44, 86.52]	5.52 [4.41, 6.63]	6.98 [5.20, 8.76]	4.62 [2.48, 6.76]	5.71 [4.39, 7.03]
3D U-Net	CNN	90.52 [89.20, 91.84]	85.80 [83.56, 88.04]	81.35 [78.12, 84.58]	85.89 [84.00, 87.78]	5.10 [4.07, 6.13]	6.25 [4.61, 7.89]	3.84 [1.87, 5.81]	5.06 [3.85, 6.27]
nnU-Net-style 3D U-Net	CNN	91.65 [90.43, 92.87]	88.42 [86.42, 90.42]	84.76 [81.88, 87.64]	88.28 [86.65, 89.91]	4.25 [3.32, 5.18]	4.85 [3.36, 6.34]	2.92 [1.17, 4.67]	4.01 [2.92, 5.10]
*Transformer-based methods*									
TransUNet	Hybrid	90.38 [89.05, 91.71]	85.95 [83.74, 88.16]	81.62 [78.43, 84.81]	85.98 [84.12, 87.84]	5.15 [4.10, 6.20]	6.18 [4.56, 7.80]	3.75 [1.82, 5.68]	5.03 [3.85, 6.21]
Swin-Unet	Pure TF	90.95 [89.67, 92.23]	86.48 [84.34, 88.62]	82.50 [79.42, 85.58]	86.64 [84.84, 88.44]	4.88 [3.89, 5.87]	5.74 [4.20, 7.28]	3.42 [1.58, 5.26]	4.68 [3.56, 5.80]
UNETR	Hybrid	91.50 [90.27, 92.73]	88.65 [86.64, 90.66]	84.20 [81.28, 87.12]	88.12 [86.48, 89.76]	4.12 [3.18, 5.06]	**4.35** [**2.85, 5.85**]	**2.40** [**0.63, 4.17**]	3.62 [2.51, 4.73]
NeuroSPFNet (ours)	Ours	**91.94** [**90.70, 93.18**]	**88.84** [**86.85, 90.83**]	**85.38** [**82.52, 88.24**]	**88.72** [**87.10, 90.34**]	**3.78** [**2.85, 4.71**]	4.48 [2.99, 5.97]	2.48 [0.74, 4.22]	**3.58** [**2.46, 4.70**]
nnFormer [11,36]	Transformer	91.46 [N/A]	87.42 [N/A]	82.22 [N/A]	87.03 [N/A]	10.15 [N/A]	9.59 [N/A]	16.78 [N/A]	12.17 [N/A]
Swin UNETR [23]	Transformer	92.70 [N/A]	87.60 [N/A]	85.30 [N/A]	88.53 [N/A]	4.74 [N/A]	15.31 [N/A]	16.33 [N/A]	12.13 [N/A]

**Table 3:** Component-ablation study of NeuroSPFNet. Score cells report mean [95% confidence interval] over the unified 251-patient evaluation set. HD95 values are in millimeters. Boldface denotes the best point estimate in this table.

Configuration	Primary Effect	CVLPC	FEBM	MLDFM	LSBCL	WT Dice	TC Dice	ET Dice	Mean Dice	Δ Dice	Mean HD95	Δ HD95
Baseline	Reference model	–	–	–	–	90.12 [88.75, 91.49]	84.85 [82.46, 87.24]	80.52 [76.94, 84.10]	85.16 [83.30, 87.02]	–	5.24 [3.95, 6.53]	–
+CVLPC	TC/ET discrimination	✓	–	–	–	90.65 [89.34, 91.96]	86.72 [84.55, 88.89]	83.15 [79.98, 86.32]	86.84 [85.10, 88.58]	+1.68	4.95 [3.74, 6.16]	−0.29
+CVLPC+FEBM	Boundary quality	✓	✓	–	–	90.58 [89.30, 91.86]	87.25 [85.14, 89.36]	84.31 [81.26, 87.36]	87.38 [85.68, 89.08]	+0.54	4.28 [3.14, 5.42]	−0.67
+CVLPC+FEBM+MLDFM	WT/TC scale fusion	✓	✓	✓	–	91.30 [90.04, 92.56]	87.95 [85.90, 90.00]	84.78 [81.82, 87.74]	88.01 [86.36, 89.66]	+0.63	4.16 [3.03, 5.29]	−0.12
Full NeuroSPFNet	Dice-boundary balance	✓	✓	✓	✓	**91.94** [**90.70, 93.18**]	**88.84** [**86.85, 90.83**]	**85.38** [**82.52, 88.24**]	**88.72** [**87.10, 90.34**]	+0.71	**3.58** [**2.46, 4.70**]	−0.58

**Table 4:** Semantic prior source ablation for CVLPC. Score cells report mean [95% confidence interval] over the unified 251-patient evaluation set. HD95 values are in millimeters. Boldface denotes the best point estimate in this table.

Prior Source	Frozen	WT Dice	TC Dice	ET Dice	Mean Dice	Mean HD95
None	–	90.12 [88.75, 91.49]	84.85 [82.46, 87.24]	80.52 [76.94, 84.10]	85.16 [83.30, 87.02]	5.24 [3.95, 6.53]
Random embedding	No	90.15 [88.79, 91.51]	84.92 [82.54, 87.30]	80.59 [77.02, 84.16]	85.22 [83.37, 87.07]	5.23 [3.95, 6.51]
Learned embedding	No	90.32 [88.98, 91.66]	85.46 [83.13, 87.79]	81.41 [77.94, 84.88]	85.73 [83.93, 87.53]	5.10 [3.84, 6.36]
CLIP text encoder	Yes	90.50 [89.17, 91.83]	85.88 [83.59, 88.17]	80.60 [77.17, 84.03]	85.66 [83.84, 87.48]	5.18 [3.91, 6.45]
BioClinicalBERT	Yes	90.54 [89.22, 91.86]	86.26 [84.02, 88.50]	82.13 [78.80, 85.46]	86.31 [84.55, 88.07]	5.01 [3.78, 6.24]
BioMedCLIP	Yes	**90.65** [**89.34, 91.96**]	**86.72** [**84.55, 88.89**]	**83.15** [**79.98, 86.32**]	**86.84** [**85.10, 88.58**]	**4.95** [**3.74, 6.16**]

**Table 5:** Frequency enhancement strategy ablation. Score cells report mean [95% confidence interval] over the unified 251-patient evaluation set. HD95 values are in millimeters. Boldface denotes the best point estimate in this table.

Strategy	Mean Dice	Mean HD95	Parameter Increase
None	86.84 [85.10, 88.58]	4.95 [3.74, 6.16]	0
Sobel edge enhancement	87.05 [85.34, 88.76]	4.40 [3.24, 5.56]	Minimal
High-frequency residual	87.47 [85.78, 89.16]	4.52 [3.35, 5.69]	Small
FFT frequency gating	**87.56** [**85.88, 89.24**]	**4.15** [**3.03, 5.27**]	Moderate but acceptable

**Table 6:** Loss function ablation for LSBCL. Score cells report mean [95% confidence interval] over the unified 251-patient evaluation set. HD95 values are in millimeters. Boldface denotes the best point estimate in this table.

Loss Setting	WT Dice	TC Dice	ET Dice	Mean Dice	Mean HD95
Dice + cross-entropy	91.30 [90.04, 92.56]	87.95 [85.90, 90.00]	84.78 [81.82, 87.74]	88.01 [86.36, 89.66]	4.16 [3.03, 5.29]
Dice + cross-entropy + boundary loss	91.62 [90.37, 92.87]	88.12 [86.09, 90.15]	84.80 [81.86, 87.74]	88.18 [86.54, 89.82]	3.70 [2.62, 4.78]
Dice + cross-entropy + semantic loss	91.35 [90.10, 92.60]	88.72 [86.72, 90.72]	85.31 [82.43, 88.19]	88.46 [86.83, 90.09]	3.92 [2.83, 5.01]
Full LSBCL	**91.94** [**90.70, 93.18**]	**88.84** [**86.85, 90.83**]	**85.38** [**82.52, 88.24**]	**88.72** [**87.10, 90.34**]	**3.58** [**2.46, 4.70**]

**Table 7:** Computational efficiency comparison. Boldface denotes the best mean Dice point estimate in this table.

Method	Params (M)	GFLOPs	Inference Time (ms/volume)	Mean Dice
U-Net	31.04	65.20	185	83.75
Swin-Unet	27.15	58.40	210	86.64
TransUNet	105.32	112.50	380	85.98
nnU-Net-style 3D U-Net	30.85	72.80	245	88.28
UNETR	92.64	95.20	340	88.12
NeuroSPFNet (Ours)	42.58	78.60	265	**88.72**

## Data Availability

The 1251 labeled BraTS 2021 cases used for model development are available through the official BraTS 2021 Synapse portal (https://www.synapse.org/Synapse:syn25829067/wiki/626944, accessed on 28 July 2026), subject to the benchmark terms and data use agreement. The 219 label-withheld challenge cases were not used to calculate the locally reported metrics.

## Abbreviations

MRI	Magnetic resonance imaging
BraTS	Brain Tumor Segmentation benchmark
VLM	Vision-language model
CNN	Convolutional neural network
FFT	Fast Fourier transform
CE	Cross-entropy
PLM	Pretrained language model
WT	Whole tumor
TC	Tumor core
ET	Enhancing tumor
ED	Edema
CVLPC	Clinical Vision–Language Prior Component
FEBM	Frequency-Enhanced Boundary Module
MLDFM	Multi-Scale Lesion-Dominated Fusion Module
LSBCL	Lesion-Semantic Boundary Consistency Loss
HD95	95th percentile Hausdorff distance
